# Progranulin and Its Related MicroRNAs after Status Epilepticus: Possible Mechanisms of Neuroprotection

**DOI:** 10.3390/ijms18030490

**Published:** 2017-02-24

**Authors:** Peter Körtvelyessy, Tessa Huchtemann, Hans-Jochen Heinze, Daniel M. Bittner

**Affiliations:** 1 Department of Neurology, University Hospital Magdeburg, 39210 Magdeburg, Germany; Tessa.huchtemann@med.ovgu.de (T.H.); Hans-Jochen.heinze@med.ovgu.de (H.-J.H.); daniel.bittner@med.ovgu.de (D.M.B.); 2 German Center for Neurodegenerative Diseases, 39120 Magdeburg, Germany; 3 Leibniz Institute for Neurobiology, 39118 Magdeburg, Germany

**Keywords:** progranulin, status epilepticus, neuroprotection, epilepsy, neurorehabilitation

## Abstract

The current knowledge about neuroprotective mechanisms in humans after status epilepticus is scarce. One reason is the difficulty to measure possible mediators of these neuroprotective mechanisms. The dawn of microRNA detection in the cerebrospinal fluid (CSF) and the recent advancements in measuring proteins in the CSF such as progranulin, which is, e.g., responsible for neurite outgrowth and limiting exceeding neuroinflammatory responses, have given us new insights into putative neuroprotective mechanisms following status epilepticus. This should complement the animal data. In this review, we cover what is known about the role of progranulin as well as the links between microRNA changes and the progranulin pathway following status epilepticus in humans and animals hypothesizing neuroprotective and neurorehabilitative effects. Progranulin has also been found to feature prominently in the neuroprotective processes under hypoxic conditions and initiating neurorehabilitative processes. These properties may be used therapeutically, e.g., through drugs that raise the progranulin levels and therefore the cerebral progranulin levels as well with the goal of improving the outcome after status epilepticus.

## 1. Introduction

Status epilepticus (SE) is a devastating and common acute disease in neurology and emergency care [1]. During convulsive status epilepticus, and possibly also during other forms of status epilepticus, hypoxic and inflammatory stress are initiated together with microglia and macrophage activation [2,3]. This stress induces several cerebral processes resulting in severe neuronal damage and therefore worsening the clinical outcome. Especially, the duration of the status epilepticus determines the clinical outcome after status epilepticus [2,4]. On the other hand, these processes initiate neuronal repair and probably initiate neuroprotective mechanisms as well.

Progranulin (PGRN) is a protein with various properties in the central nervous system (CNS) including important roles in repair mechanisms as well as responses to hypoxemia and limiting neuroinflammation. As a response to status epilepticus, PGRN is secreted by activated microglia as part of neuroregeneration and probably neuroprotection. This has been shown in rats [5] and presumably also in humans [6], though further research is needed here. The aim of this review is to provide an overview of the data on PGRN and status epilepticus including the proteins and miRNA strongly related to PGRN hypothesizing the role of these proteins in the neuroregenerative and neuroprotective mechanisms during and after status epilepticus.

## 2. Progranulin

Progranulin is a 68.5-kDa precursor glycoprotein with 7.5 granules which undergoes proteolytic processing; each cleaved granulin or Progranulin itself has supposedly different functions [7,8,9]. It is built by a quarternary structure of β-Hairpins stacks [10]. The main proteolytic enzymes cleaving the granules are matrix-metallo-proteinases (MMP-12 and MMP-14) [7], one of the inhibitors of PGRN proteolysis is the secretory leukocyte protease inhibitor (SLPI) [11]. PGRN is ubiquitously expressed in all kinds of tissues. There is growing evidence that PGRN is regulated differently in the CNS compared to other tissues and in plasma [12,13]. In the CNS, PGRN is secreted by neurons and microglia throughout the brain with emphasis on frontal, hippocampal, entherorhinal areas and in the cerebellum [14,15]. PGRN has a neuroprotective role via promoting neurite outgrowth in neurons [16,17], and as a promoter of neuronal survival via Sortilin and P75_NTR_ [7,18,19,20] It also curbs the cytokine expression by CD11b positive macrophages and CD11b positive microglia [11]. PGRN expression in the CNS is elevated following hypoxia [21,22], neuroinflammation [23], trauma [24,25,26], stroke [27] and status epilepticus [5,6]. Up until now three PGRN-receptors have been identified, each with different functions. The tumor-necrosis-factor receptor 1 (TNFR1) could play a major role in PGRN-influenced neuroinflammation [23,28]. The sortilin-receptor 1 (SORT1) is part of many pathways [18], e.g., Sortilin is expressed in activated microglia after injury. Very recently, the ephrin receptor kinase 2A was discovered as another receptor of PGRN [29].

PGRN became a matter of interest to the neuroscientific community after it was discovered that Granulin mutations cause tau-negative fronto-temporal dementia (FTD) [30,31,32]. Up to date, 70 GRN mutations in 233 families are described [32,33]. Since then, various roles and expression patterns of PGRN in the brain have been discovered. Low CSF-PGRN levels have been found in FTD, with or without Granulin mutation [34,35], or certain forms of neuronal ceroid lipufuscinosis (NCL) [36]. The GRN gene is located on chromosome 17q21. Approximately 5%–11% of sporadic FTD-cases and 20% of familial FTD-cases are caused by GRN mutations [36]. These 17q21-linked FTD are all autosomal-dominant, homozygous GRN mutations resulting in haploinsufficiency. On the other hand, null mutations in the GRN gene can cause NCL Typ 11 resulting in a lysosomal storage deficiency and accumulation of lipoid clusters in neurons [36,37]. These NCL patients suffer from severe epilepsy, progressive visual loss and neuropsychiatric disorders leading to a shorter life span. Patients having a GRN mutation show a variety of different diseases ranging from FTD, to NCL to a motoneurondisease [37,38]. Both, GRN mutation positive and GRN mutation negative FTD patients share a Tar-DNA-Protein-43 (TDP-43) mediated pathomechanism [39]. However, the direct link between TDP-43-mediated mechanism and PGRN is still missing [8]. Since the initial discovery of its significance in FTD and then NCL, the role of PGRN has been further examined in other diseases and in various injury modalities. Xu et al. (2011) conducted several in vitro experiments where cell cultures, either with or without PGRN, were exposed to oxidative stress. Their findings proved a significant role of PGRN in improving cell survival and further showed that the neuroprotective effect depended on the toxic agent they used [40]. Jackman et al. (2013) showed that PGRN-deficient mice suffer a significantly larger ischemic injury and increased hemorrhage after transient focal ischemia than wild type mice [27]. Contrary to previous theories that suspected post-ischemic inflammation or hemodynamic dysfunction as a reason for an increased ischemic injury, Jackman et al. discovered altered tight junctions and a consecutive breakdown of the blood brain barrier (BBB) in PGRN-deficient mice [27]. This mechanism is especially interesting in light of the fact that the BBB and changes in its permeability have been implicated in epileptogenesis for several decades [41].

### 2.1. Progranulin in Neuroinflammation and Hypoxia

Recently, PGRN has been identified as an important factor in the response to immunological stress or hypoxic conditions. Increased PGRN levels are caused by the response of the CNS to immunological stress induced by various triggers, e.g., lipopolysaccharides or brain trauma [24,25,26] PGRN is assumed to have anti-inflammatory properties when secreted by microglia. Potentially neurotoxic cytokines have been shown to be overexpressed in progranulin-deficient mice [25,42]. In addition, macrophages in PGRN-deficient mice produce significantly less Interleukin-10 and more pro-inflammatory cytokines such as Interleukin-6 CXCL-1 or tumor-necrosis-factor [42]. Furthermore, the endocytotic functions of macrophages and microglia are significantly changed by PGRN levels [11] and it serves as a chemoattractant for microglia and stimulates their endocytotic activity [43]. Exposing PGRN-deficient mice to 1-methyl-4-(2′-methylphenyl)-1,2,3,6-tetrahydrophine (MPTP), which causes acute inflammation and cell death, caused a higher loss of neurons and increased inflammatory activity by microglia compared to wildtype mice [23]. Treating PGRN-deficient microglia with lipopolysaccharides and Interferone-γ resulted in higher expression of pro-inflammatory cytokines (interleukin 1b, interleukin-6, and tumor-necrosis-factor-α), similar to the mechanism in PGRN-deficient macrophages described above.

Several experiments by the group of Nishihara Masuki further illuminated the role of PGRN after acute immunological stressors [24,25,26]. PGRN seems to protect hippocampal neurons in acute immunological stress by probably promoting the mammalian target of rapamycin (mTOR) pathway [44]. The exact effect of PGRN on the lysosomal metabolism could not be clarified, but it seems that PGRN is at least co-localized in the lysosomes of activated microglia after traumatic brain injury [34]. This lysosomal metabolism of PGRN in microglia seems to play a prominent role because both gene loci are activated at the same time after traumatic brain injury. In summary, PGRN most likely plays an important part in the regulation of the immunological microglial response under various injury modalities.

### 2.2. Progranulin in Epilepsy

Zhu et al. investigated the role of PGRN in mice after status epilepticus [5]. In status epilepticus, microglial activation in the hippocampus, together with an increased infiltration of the hippocampus, has been seen. Zhu et al. found increased GRN-mRNA and PGRN-levels after pilocarpine-induced status epilepticus. The increase in PGRN-levels in the brain lysates was delayed and peaked 48–96 h after the status epilepticus occurred. A higher concentration of CD11 positive macrophages and microglia were also detected in the brain lysates and in a double-immunostaining of CD11 and PGRN), pointing at a PGRN-associated increase of these CD11 positive cells. The same effect was observed after PGRN was injected in the hippocampus right after the status epilepticus. It is not entirely clear though if this activation is due to the injection itself or an effect of PGRN. However, this injection of non-endogenous “early” PGRN did not prevent neuronal cell death in the dentate gyrus.

These data in rats prompted us to measure PGRN in the CSF of humans after status epilepticus and after single grand-mal seizures [6]. We found a significant increase of PGRN in the CSF after status epilepticus. There was also a significant and delayed increase of the CSF-PGRN levels in humans after a single grand-mal seizure, thus a delayed increase in PGRN levels seems not to be limited to the status epilepticus but to a single grand mal seizure as well. The study was underpowered to detect significant differences between grand-mal seizures and status epilepticus. The mechanism behind this delayed increase in PGRN remains elusive. As reported above though, PGRN is expressed under neuroinflammatory and hypoxic conditions. Both processes also occur in status epilepticus and probably during epileptogensis [2,3]. Analogous to the delayed increase following a status epilepticus, a delayed increase of PGRN has been detected in the hippocampal lysosomes after treatment with lipopolysaccharide [44]. The expression of PGRN mRNA first increased significantly after 24 h and even more after 48 h. On the other hand PGRN CSF-levels are very high in patients suffering from viral or bacterial meningitis right at beginning of the symptoms and there are no signs of a delayed increase although thorough investigations are lacking (personal communication Dr. Bittner). These results may be due to different pathways of neuroinflammation after a seizure or status epilepticus and in acute infections.

Under hypoxic conditions, PGRN and microRNAs (miRNA) possibly controlling PGRN have been shown to be upregulated in neuroblastoma cells [22]. When measuring PGRN messengerRNA after a hypoxic stress, a delayed increase with a peak at Day 4 was seen [22]. This delay could be due to neuroprotective and anti-inflammatory mechanisms mediated partly by the PGRN pathway. Interestingly, the disruption of the BBB is a parameter that also occurs with a delay after status epilepticus [45], though further research has to be conducted to connect the delay of PGRN-increase and the delayed BBB disruption.

In conclusion, investigating PGRN levels in rats and human CSF reveals a delayed increase after status epilepticus as well as under various other hypoxic and inflammatory conditions. Further studies should enlighten the reasons for the delay and associated mechanisms.

## 3. Progranulin and MicroRNA

### 3.1. Role of miRNA in the Progranulin Pathway

miRNA are small non-coding RNA playing an important role in the post-transcriptional modification of mRNA and therefore in protein regulation [46,47]. This step between gene transcription and protein translation allows for a swift and dynamic response to the various stressors that can potentially cause major damage to the fragile brain tissue (hypoxia, trauma, and infarction). Status epilepticus causes inflammation, synaptic remodeling and neurogenesis with increased proliferation, cell migration and deregulated differentiation [3]. Numerous miRNA have been found to be elevated after status epilepticus, while the expression of a few other miRNA have been shown to be reduced [46,48]. Inhibiting some of the elevated miRNA after status epilepticus has already been tested in vivo (e.g., miRNA-134 [49]). The results have shown seizure-reducing and neuroprotective effects mostly through modification of the inflammatory response as well as effects on cell death, cell migration, proliferation and differentiation. The exact proteins that are targeted by miRNA are still mostly unknown though, as are the miRNA that control the proteins and pathways with major roles in neuroprotection. The pathway of PGRN or PGRN itself is influenced by several miRNA including miRNA-132, miRNA-659-3p, miRNA-107 and miRNA-9.

### 3.2. miRNA-659-3p

The majority of miRNA with links to various diseases, as e.g., miRNA-132, have been found through profiling miRNA that are either up- or down-regulated after certain traumatic events (status epilepticus, traumatic brain injury) or in certain neurodegenerative disorders such as FTD and Alzheimer’s Disease (AD). Another approach has been to analyze miRNA that control the expression of proteins that are directly involved in the pathophysiology of a disease. For FTD, this happens to be PGRN. Since the majority of miRNA control genes through modification at the 3′UTR-end, Piscobo et al. [22] analyzed the 3′UTR-end of the *GRN*-gene and found two possible binding sites for miR-659-3p. Further tests showed that an increase of miRNA-659-3p caused a decreased expression of GRN-RNA, this suggests that binding of miRNA-659-3p with the GRN-RNA inhibits its translation. The same workgroup already demonstrated before, that hypoxic conditions in cells cause an increase of PGRN expression [21]. In their more recent paper this theory has been confirmed in a rat model [22]. It has also shown that miR-659-3p decreases under hypoxic conditions while the concentration of PGRN increases with a delay, probably similar to the PGRN levels seen in humans and rats. As mentioned above (Section 2.1), hypoxic conditions are assumed to be part of the devastating mechanisms involved in the brain of status epilepticus patients. Therefore, it is tempting to speculate that markers of hypoxic conditions such as Hypoxia-inducible-Factor1 or miRNA-659-3p may also participate in the pathomechanisms involved during and after status epilepticus but more studies are needed to prove this.

### 3.3. miRNA-107

GRN has been found in a high-throughput experimental miRNA assay to be the strongest target for miRNA-107 [50]. Profiling miRNA in the human cerebral cortex has shown a far higher level of miRNA-107 than, e.g., miRNA-659-3p. MiRNA-107 has been studied in AD [51], in a mouse model of traumatic brain injury (TBI) as well as in monoclonal blood cells of young and older individuals. In AD as well as in the TBI mouse model there has been a marked decrease of miRNA-107, the cells of older individuals have shown a decreased expression of miRNA-107 as well [52]. The reduced expression of miRNA-107 causes an up-regulation of PGRN in the affected cells (though this was not confirmed in Piscobo et al., 2016 [22]). Corresponding to the mechanism for miR-659-3p, the correlation between decreased levels of miRNA-107 and higher levels of PGRN in the affected cells indicates a neuroprotective mechanism. Thus far miRNA-107 has mostly been studied in the two diseases above, and no direct link to SE or FTD has been found yet.

### 3.4. miRNA-132

The miRNA-132 cluster consists of miRNA-132a, miRNA-132* and miRNA-212. One of the effects of the low expression of miRNA-132/212 is that it leads to an up-regulation of a transmembrane protein (TMEM106B), which disturbs the PGRN pathway and therefore has a part in increasing the likelihood of FTD. MiRNA-132 is one the best researched miRNA as it is the most consistently up-regulated miRNA following a SE [46]. Inhibition of miRNA-132 resulted in a reduction of the number of seizures in mice after pilocarpine-induced SE [49]. Over-expression of miRNA-132 has also shown to contribute to epileptogenesis through neuronal and dendritic sprouting as well as increased migration [53]. This may have negative effects after a SE since it could increase the size of epileptogenic lesions, but on the other hand suggests a rather important role of miRNA-132 in the nervous system. What we know so far about the PGRN pathway in relation to the miRNA-132-cluster and the role of miRNA-132 in neurodegenerative diseases also hints at important neuroprotective effects of miRNA-132. For example, a reduced expression of the miRNA-132-cluster has been verified for two forms of dementia, FTD and AD [54]. For FTD, Chen-Plotkin et al. (2012) have demonstrated that all three members of the miRNA-132-cluster have shown <50% expression in FTD-brains compared to normal controls [55]. This research also indicates a more general role of miRNA-132, one that is not specific to SE. As reported above, blocking miRNA-132 following a SE has shown to cause short-term positive effects. Thus far no studies have examined possible long-term effects, but considering the role of miRNA-132 in FTD blocking miRNA-132 and consecutive down-regulation of PGRN may also have a harmful effect on the brain after status epilepticus.

### 3.5. miRNA-9

The majority of research has been done on the miRNA mentioned above, but there are other possible candidates that might further enlighten the role of PGRN and the neuroprotective mechanisms following a SE. One of these is miRNA-9; its expression has been found to be reduced in induced-pluripotent-stem-cell-derived neurons of FTD-patients with *TDP-43* mutations [56]. TDP-43 is responsible for stabilizing GRN-RNA [57]. On the other hand, and similarly to miRNA-132, in epilepsy (animal model and human) miRNA-9 has shown to be elevated, possibly being proepiletogenic through inhibiting the *NFkB1*-transcript and therefore increasing inflammation [58] as well as increasing migration [59]. We therefore suggest to further look at this interesting miRNA in relation to neurodegeneration and neuroinflammation.

## 4. Progranulin and Therapeutic Intervention

Initiating neuroprotective mechanisms right at the beginning of the treatment of a status epilepticus could significantly improve the clinical outcome. Supporting neuroprotection in general has been tried by some groups [60,61,62] through targeting several receptors and other proteins (purinergic P2X7, Ghrelin, PSD95) with moderate effects in terms of neuroprotection. With its suspected neuroprotective properties, raising cerebral PGRN levels may contribute to neuroprotection and facilitate regeneration. As mentioned above PGRN levels are significantly decreased in FTD patients with a loss-of-function mutation in the *GRN* gene and probably also in FTD patients without *GRN* mutations [34,63]. Therefore, therapeutic interventions to raise the PGRN levels are now in the focus of treatment strategies for these patients. In this context the identification of PGRN modulators is of paramount importance to find possible drugs as candidates for the treatment of PGRN-related neurodegenerative diseases [63] and now as possible protective treatment in status epilepticus. A first in vivo experiment has shown that although exogenous PGRN was activating microglia/macrophages, no positive effect on neuronal survival in the dentate gyrus has been proven when PGRN was given at the same time the status epilepticus was induced [5]. Outside of status epilepticus four studies have been conducted that tested substances that might raise PGRN levels. In cells derived from patients with FTD and PGRN, null mutation a number of alkalizing reagents, e.g., bepridil or chloroquin, have been identified that increased PGRN levels [64]. Another study screened for chemical substances that might increase GRN mRNA and protein levels in GRN haploinsufficient cells and found suberoylanilide hydroxamic acid, which is a histone deacetylase inhibitor and is already approved by the Food and Drug Association [65]. Nimodipine on the other hand has not been proven to be effective in an open-label study on eight patients [66]. Lastly, Bittner et al. have shown that chloroquin raised the CSF-PGRN levels in three of three FTD-patients, which is paralleled by an improvement in cognitive abilities [67]. Overall targeting the PGRN pathways seems to be an interesting approach to support endogenous neuroregenerative potential. Further studies are needed to explore other strategies and substances that raise PGRN levels in the brain.

## 5. Conclusions

PGRN and its related pathway seem to embody interesting targets with the potential of improving the outcome of patients suffering from a status epilepticus [68]. Especially, miRNA related to the PGRN pathway that are easy to turn off could be an interesting target, although direct evidence is not published yet. The delay in the increase of PGRN and of some miRNA strongly related to the metabolism of PGRN support the hypothesis that PGRN is part of the neuronal repair mechanisms and not part of the acute neuroinflammatory response caused by status epilepticus. However, it is not clear whether hypoxia contributes additionally or solely to the PGRN level increase. Low CSF-PGRN levels correlate with increased neuronal cell death as in neurodegenerative diseases, high CSF-PGRN levels could be an indicator of neuroregeneration and neuronal cell survival, as seen in several studies (see also Section 2). Studies on raising the PGRN level in the CNS are currently underway. This approach might enable new clinical treatments for neurodegenerative diseases and possibly status epilepticus as well.

Despite a wealth of new findings on neuroprotection and neuronal repair in status epilepticus, stopping the status epilepticus as soon as possible is still crucial for improving the clinical outcome [2]. The goal in the future should be to initiate neuroprotective mechanisms and neuronal repair parallel to the treatment of the status epilepticus.

## Abbreviations

AD	Alzheimer Disease
BBB	Blood brain barrier
CNS	Central nervous system
CSF	Cerebrospinal fluid
FTD	Tau-negative fronto-temporal Dementia
kDa	Kilo Dalton
miRNA	microRNA
mRNA	Messenger RNA
NCL	Neuronal lipofuscinosis
PGRN	Progranulin
SE	Status epilepticus
